# Extracellular Vesicle (EV) Proteomics in Corneal Regenerative Medicine

**DOI:** 10.3390/proteomes13040049

**Published:** 2025-10-03

**Authors:** Zohreh Arabpour, Hanieh Niktinat, Firouze Hatami, Amal Yaghmour, Zarife Jale Yucel, Seyyedehfatemeh Ghalibafan, Hamed Massoumi, Zahra Bibak Bejandi, Majid Salehi, Elmira Jalilian, Mahmood Ghassemi, Victor H. Guaiquil, Mark Rosenblatt, Ali R. Djalilian

**Affiliations:** 1 Department of Ophthalmology and Visual Science, University of Illinois, Chicago, IL 60612, USA; arabpour@uic.edu (Z.A.); jalilian@uic.edu (E.J.);; 2 Department of Tissue Engineering, School of Medicine, Shahroud University of Medical Sciences, Shahroud 3614773955, Iran

**Keywords:** extracellular vesicle, proteomics, corneal regeneration, mesenchymal stem cells, extracellular vehicles priming

## Abstract

Corneal regeneration has gained growing interest in recent years, largely due to the limitations of conventional treatments and the persistent shortage of donor tissue. Among the emerging strategies, extracellular vehicles (EVs), especially those derived from mesenchymal stromal cells (MSCs), have shown great promise as a cell-free therapeutic approach. These nanoscale vesicles contribute to corneal healing by modulating inflammation, supporting epithelial and stromal regeneration, and promoting nerve repair. Their therapeutic potential is largely attributed to the diverse and bioactive proteomic cargo they carry, including growth factors, cytokines, and proteins involved in extracellular matrix remodeling. This review presents a comprehensive examination of the proteomic landscape of EVs in the context of corneal regenerative medicine. We explore the biological functions of EVs in corneal epithelial repair, stromal remodeling, and neurodegeneration. In addition, we discuss advanced proteomic profiling techniques such as mass spectrometry (MS) and liquid chromatography–mass spectrometry (LC-MS/MS), which have been used to identify and characterize the protein contents of EVs. This review also compares the proteomic profiles of EVs derived from various MSC sources, including adipose tissue, bone marrow, and umbilical cord, and considers how environmental cues, such as hypoxia and inflammation, influence their protein composition. By consolidating current findings, this article aims to provide valuable insights for advancing the next generation of cell-free therapies for corneal repair and regeneration.

## 1. Introduction

**Figure 1:**
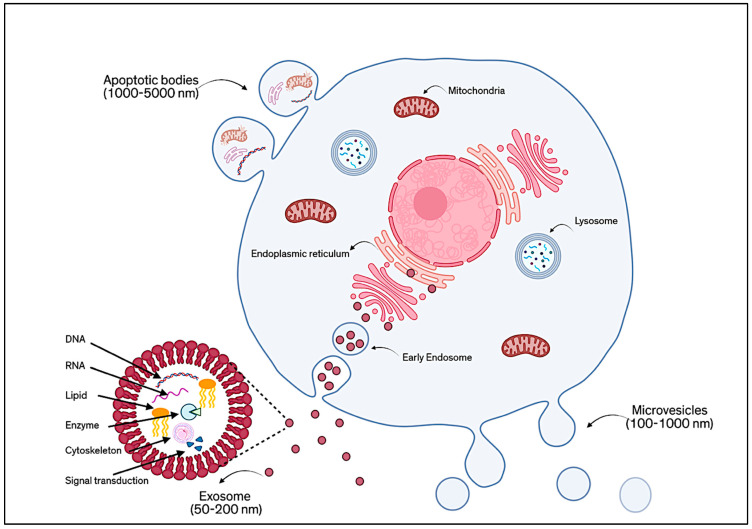
Schematic of the classification of EVs based on their mode of formation.

EVs have gained significant attention as powerful, cell-free tools in regenerative medicine, especially those derived from MSCs [1]. These small, naturally secreted particles, surrounded by a protective lipid bilayer, act as messengers between cells by carrying a wide variety of biologically active components, including proteins, lipids, RNA, DNA, cytokines, and growth factors. EVs are generally classified according to their mode of formation, as shown in Figure 1. There are three main types: ectosomes (or microvesicles), exosomes, and apoptotic EVs [2]. Ectosomes (about 50 nm to 1 µm) form directly from the outward budding of the cell membrane. Exosomes (typically 30–150 nm) are created inside the cell through an endosomal process and are released when multivesicular bodies fuse with the plasma membrane [3]. Apoptotic EVs (100 nm to 5 µm) are released when a cell undergoes programmed death [4]. However, because these different types of EVs can be difficult to distinguish only by size or surface markers, the MISEV 2023 guidelines recommend using the general term “EVs”, unless their origin and features are clearly defined. Broadly speaking, EVs are understood as non-replicating, lipid-bound particles released by cells [5].

Among these, exosomes have become a focal point in research due to their relatively uniform structure and well-documented functions. Exosomes are tiny, spherical vesicles surrounded by a bilayer membrane rich in lipids such as cholesterol, ceramides, and sphingomyelin. Their membranes also feature proteins like tetraspanins (e.g., CD9, CD63, CD81), which serve as characteristic markers. In addition, exosomes may carry glycoproteins, adhesion molecules, and antigen-presenting proteins [6]. The content of exosomes, their “cargo”, is shaped by the type of cell they come from, the cell’s health or stress level, and how the exosomes are made. Compared to the parent cells, exosomes are often enriched with specific proteins like cytoskeletal elements, heat shock proteins, and tetraspanins, and they also contain genetic material, metabolic products, and signaling molecules [7].

These complex and dynamic features explain why EVs, particularly exosomes, hold such promise for tissue repair, including in the cornea. In recent years, research has shown that EVs from MSCs can reduce inflammation, support corneal epithelial healing, encourage regeneration in the stroma, help remodel damaged matrix, and even assist in nerve repair. Much of this regenerative ability is believed to be tied to the proteins and other molecules packaged within the vesicles [8].

Recent studies have demonstrated that EVs contribute directly to corneal regeneration by regulating key cellular processes. EVs can promote epithelial wound closure, modulate stromal fibrosis, stimulate keratocyte proliferation, support extracellular matrix remodeling, and enhance nerve regeneration. These effects are largely mediated through the transfer of bioactive proteins, miRNAs, and signaling molecules that orchestrate cell proliferation, differentiation, and immune modulation in a context-dependent manner [8,9,10]. Notably, EVs can influence corneal cells via multiple mechanisms, including activation of PI3K/Akt and MAPK signaling, inhibition of myofibroblast differentiation, and modulation of angiogenesis, thereby facilitating coordinated tissue repair [11].

It is important to consider the health of the source, as emphasized by the comparison between non-diabetic (N) and diabetic (DM) limbal stromal cell (LSC)-derived Exos. N-Exos promote wound healing in N-LSCs more effectively than DM-Exos. Phosphorylated/activated p-ERK1/2 is expressed during the healing process in wounded LSCs treated with N-Exos, whereas this is not observed with DM-Exos, highlighting the role of cell-signaling in the process [11].

Advanced analytical techniques like liquid chromatography–tandem mass spectrometry (LC-MS/MS) have made it possible to dive deep into the protein makeup of EVs. This type of proteomic profiling has shown that EVs carry a selective and often cell-specific cargo that reflects not only their cell of origin, but also the conditions under which they were produced [9].

Interestingly, EVs from various corneal cell types like epithelial cells, keratocytes, fibroblasts, and myofibroblasts have been shown to carry unique protein profiles that mirror their functional roles in corneal health, repair, or scarring. Similarly, EVs derived from different MSC sources (e.g., bone marrow, adipose tissue, or umbilical cord) also display distinct proteomic patterns. These patterns can shift further depending on environmental factors like inflammation, hypoxia, or mechanical stress [10].

In this review, we take a close look at the proteomic content of EVs and how it supports their application in corneal regeneration. By exploring how EVs are classified, how they are formed, and what they carry, we aim to highlight their therapeutic potential. Special attention is given to the proteomic signatures from different EV sources and their relevance in promoting effective and targeted healing of the cornea.

## 2. EVs in Corneal Regeneration

The cornea is the most superficial and susceptible part of the eye to damage by mechanical trauma, chemical injury, ultraviolet radiation, and infections [12]. The corneal epithelium, Descemet’s membrane, and corneal nerves have the capacity to regenerate following injury; in contrast, the stromal layer exhibits limited regenerative potential [13]. Extracellular vesicles, with their high biological compatibility and low immunologic reactivity, are being explored as novel mediators of inflammation control and corneal tissue regeneration [14]. EVs’ regenerative capacity is largely attributed to their complex and diverse proteomic composition, enriched with growth factors, cytokines, and bioactive proteins, which play a pivotal role in modulating inflammation and influencing tissue regeneration [15]. These bioactive components contribute to epithelial regeneration, restructuring of the corneal stroma, and fostering corneal nerve regeneration.

**Figure 2:**
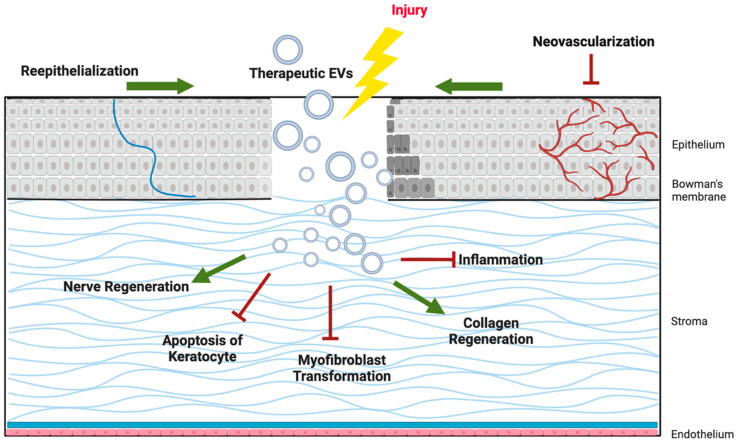
Schematic illustration of the Potential mechanisms of extracellular vesicles (EVs) in corneal regeneration.

As illustrated in Figure 2, EV cargo proteins support epithelial wound closure, suppress fibrosis in the stroma, promote axonal regeneration in corneal nerves, modulate immune responses, and regulate angiogenesis to preserve corneal transparency. These multifaceted effects highlight EVs as promising candidates for cell-free therapies in corneal regenerative medicine.

### 2.1. Epithelial Regeneration

Mechanical or chemical corneal injuries induce an inflammatory response, epithelial cell proliferation and migration, and extracellular matrix remodeling. Extracellular vesicles play a crucial role in these processes by modulating inflammation and angiogenesis, promoting epithelial regeneration, and exerting anti-apoptotic effects [16,17].

Extracellular vesicles promote corneal epithelial healing by upregulating genes associated with epithelial cell proliferation and migration. These include EGFR, MMP9, CDC42, FRS2, and CTGF, which contribute to enhancing epithelial migration [18].

Mesenchymal stem cell-derived EVs (MSC-EVs) have been shown to significantly boost epithelial regeneration both in vitro and in vivo [14]. For instance, Liu et al. demonstrated that small EVs derived from human umbilical mesenchymal stem cells (HUMSC-sEVs) are enriched in miR-21, which suppresses PTEN mRNA and activates the PI3K/Akt signaling pathway, thereby enhancing corneal epithelial proliferation and migration after injury [19]. Similarly, Wang et al. showed that MSC EVs activate the NGF/TrkA/Akt signaling axis, leading to improved epithelial wound healing in diabetic mice [20].

In addition to stem cell-derived vesicles, EVs from corneal myofibroblasts have also been implicated in epithelial repair. These EVs are enriched with proteins such as CXCL1, CXCL6, CXCL12, MMP1, MMP2, L1CAM, and LRRC15, all of which are involved in promoting epithelial cell migration, proliferation, survival, and regeneration following injury [10].

While MSC-derived EVs are the most extensively studied, other cell types also contribute to corneal regeneration via EV secretion. Corneal epithelial cell-derived EVs deliver structural proteins (keratins, desmosome components) that enhance epithelial barrier integrity and wound closure. Endothelial cell-derived EVs contribute to ECM remodeling and immune modulation, whereas cord-lining stem cell EVs exhibit a strong pro-angiogenic profile, supporting vascular repair in ischemic contexts. Platelet-derived EVs (PEVs) and cord blood EVs have also been reported to carry regenerative protein cargo, such as VEGF, PDGF, and thrombospondin-1, and may complement MSC-EVs in ocular repair. These diverse EV sources suggest that proteomic signatures are tightly linked to cell origin, underscoring the need for comparative studies that define optimal EV populations for corneal therapy [16].

Yi et al. illustrated EVs derived from human amniotic epithelial cells (hAEC-EVs) activate several key regenerative pathways, PI3K/Akt, Wnt, cadherin, ECM-receptor interaction, and focal adhesion, demonstrating high expression of proliferation markers (PCNA, Ki67) and effective restoration of epithelial structure [21]. Likewise, MSC-EVs promote corneal epithelial integrity by enhancing the expression of vinculin, ZO-1, Esrp1, and Spp1, supporting both structural restoration and repair [16,18].

**Proteomic Composition and Functional Implications:** The regenerative capacity of EVs is dictated by their cellular origin and proteomic composition, with epithelial-derived EVs offering the most targeted and potent cargo for corneal epithelial healing. Braunsperger et al. identified that limbal epithelial progenitor cell-derived sEVs (LEPC-sEVs) are enriched in structural and adhesion-related proteins such as keratins (KRT14, KRT17), desmosome components (DSG1, DSP, JUP), and extracellular matrix (ECM) regulators, including laminin-332 subunits, collagen XVII (COL17A1), and fibronectin (FN1), essential for epithelial adhesion, migration, ECM remodeling, and corneal integrity [22].

Similarly, Yeung et al. profiled EVs from multiple corneal cell types, including epithelial, keratocytes, fibroblasts, and endothelial cells, revealing cell-specific protein cargos that contribute to divergent regenerative roles. They found epithelial-derived EVs are enriched in epithelial-specific cytoskeletal proteins keratins (KRT7/8/18/19), WFDC2, and GPRC5A, which are essential for epithelial identity, barrier function, and repair. These EVs also displayed strong activation of the EIF2 signaling pathway, vital for stress-induced protein synthesis regulation. In contrast, EVs from keratocytes and fibroblasts are enriched in lipid metabolism (e.g., APOA1, DMKN) and ECM proteins (e.g., PROZ, SDC2, TMOD2), contributing indirectly to stromal support and ECM remodeling [1].

In another proteomic study, An et al. showed that EVs from MSC-conditioned media carry bioactive proteins and regulatory molecules such as fibronectin-1 (FN1), tissue inhibitor of metalloproteinases-2 (TIMP2), and transforming growth factor-beta-induced protein (TGFBI). These proteins act through signaling pathways, including PI3K/Akt, TGFβ, and focal adhesion, highlighting the central role of EV protein content in epithelial regeneration [23].

**Reprogramming and Differentiation:** EVs can influence corneal epithelial cell fate by modulating gene expression associated with stemness and differentiation. Li et al. demonstrated that EVs from adipose-derived MSCs (ADMSC-EVs) reprogram limbal epithelial cells (LECs) toward a less differentiated progenitor-like state by upregulating WNT5A, VIM (vimentin), and FN1 (fibronectin 1), while downregulating differentiation markers like KRT12 and KRT13 [24].

Beyond regenerative stimulation, EVs exert potent cytoprotective effects. They downregulate pro-inflammatory cytokines (IL-6, IL-1β, TNF-α, NF-κB) while upregulating anti-inflammatory mediators (IL-10, TGF-β), thus attenuating injury-induced inflammation, which indicates their potent immunomodulatory effects [14,16,17]. Additionally, EVs suppress apoptotic signaling by downregulating pro-apoptotic gene expression, such as P53, BAD, and Caspase-3/8, and upregulating anti-apoptotic BCL-2 levels [14,17].

Post-injury neovascularization threatens corneal transparency. EVs mitigate this by reducing expression of angiogenesis-related genes, including Vegfa, Vegfd, Flt1, Kdr, Flt4, and MMPs (MMP2, MMP9), preserving corneal avascularity and function [14]. Extracellular matrix (ECM) remodeling plays a critical role in restoring corneal structure, integrity, and function following injury. Emerging evidence highlights epithelial EVs as key mediators in modulating ECM composition and organization during corneal regeneration. Meanwhile, stromal and endothelial EVs may contribute indirectly by remodeling the ECM or modulating inflammation [1].

Further supporting this concept, small extracellular vesicles (sEVs) derived from limbal epithelial progenitor cells (LEPC-sEVs) were shown to contribute to epithelial development and ECM-receptor interactions. These vesicles deliver a specialized proteomic cargo that regulates cytoskeletal organization and supports niche homeostasis, thereby promoting efficient re-epithelialization during corneal repair [22]. Proteomic studies by An et al. also highlighted key ECM remodeling proteins (FN1, TIMP2, TGFBI) within the EV fraction of MSC-conditioned media, which were essential for cytoskeletal remodeling, cell–extracellular matrix (ECM) interactions, and epithelial healing [23].

Hu et al. demonstrated that human amniotic epithelial cell-derived EVs (hAEC-EVs) accelerate corneal re-epithelialization and preserve stromal architecture. Their cargo includes miRNAs (miR-27a/b-3p, miR-181a-5p, let-7b-5p) and proteins (FN1, TGFBI, CD44, TIMP2) that regulate key regenerative pathways such as TGF-β, PI3K/Akt, mTOR, and focal adhesion. Focal adhesion kinase (FAK) activation and upregulation of talin-1 and paxillin by EVs support cell–ECM interactions critical to epithelial repair [25].

### 2.2. Nerve Regeneration

Corneal nerve impairment can lead to various ocular surface diseases due to its role in maintaining corneal homeostasis, sensation, epithelial integrity, and tear production. Conditions such as Neurotrophic Keratopathy (NK), Dry Eye Disease (DED), Diabetic Keratopathy, Herpetic Keratitis, and post-surgical nerve injury are closely associated with corneal nerve dysfunction [26,27]. Corneal nerves have a limited and slow intrinsic ability to regenerate, and current treatments for nerve repair and regeneration often demonstrate limited effectiveness. However, studies have demonstrated that EVs, particularly those derived from MSCs, can promote corneal nerve regeneration. These EVs are enriched with neural growth factors, signaling peptides, and regulatory proteins that support nerve survival, induce axonal outgrowth, modulate immune responses, and facilitate corneal nerve regeneration following injury [8,28,29].

**Axonal Outgrowth:** Bone marrow-derived mesenchymal stem cell extracellular vesicles (BM-MSC-EVs) promote corneal nerve regeneration by enhancing neurite elongation, branching, complexity, axonal growth, and cytoskeletal remodeling in vitro [28]. This regenerative effect is driven by their cargo of bioactive proteins and neurotrophic factors. The cellular origin of EVs and their miRNA profile contents can determine their neuroregenerative effect. For instance, corneal MSC (CO-MSC) EVs are enriched in neurogenic miRNAs like hsa-miR-128-3p and hsa-miR-127-3p, which are associated with localized neuroregenerative activity. In contrast, BM-MSC EVs enriched in miRNA have systemic effects like immunoregulation and extracellular matrix reorganization. The culture environment also plays a critical role; 3D-cultured MSCs generate EVs with more uniform miRNA profiles, suggesting adaptation to their microenvironment [30].

Proteomic profiling of MSC-EVs has revealed the presence of neurotrophic growth factors such as nerve growth factor (NGF), brain-derived neurotrophic factor (BDNF), fibroblast growth factor-1 (FGF-1), glial cell-derived neurotrophic factor (GDNF), and insulin-like growth factor-1 (IGF-1). These molecules promote neuronal survival, axonal elongation, synaptic integrity, and cytoskeletal reorganization [31]. In a clinical study, Pieragostino et al. performed proteomic analysis of tears from patients with neurotrophic keratitis treated with recombinant human NGF (rhNGF). Proteomic data revealed early activation of BDNF and Versican core protein (VCAN)-mediated pathways, followed by strong IL-6 receptor signaling and MMP-9 modulation at later stages, promoting axonal remodeling and neurogenesis. These changes were confirmed by in vivo confocal microscopy, showing progressive increases in nerve fiber length, density, and branching over time [32].

Wang et al. demonstrated that adipose-derived MSC-EVs promote corneal nerve regeneration in diabetic keratopathy by activating the NGF/TrkA/Akt signaling pathway in vitro and in vivo. This pathway is a critical axis involved in neuroprotection and axonal growth, also enhancing neurotrophic support and epithelial healing [20]. Similarly, Yu et al. showed that EVs from skin-derived precursor Schwann cells (SKP-SC-EVs) activate the same NGF/TrkA/Akt cascade, leading to axonal growth, neural survival, and functional recovery. These findings underscore the potential of EVs in corneal nerve regeneration through sustained delivery of neurotrophic signals, immune modulation, and structural repair [33].

**Modulation of neuroinflammation:** Following corneal injury, neuroinflammation is initiated by local immune cells and damaged nerves, creating an inflammatory microenvironment that hinders axonal regrowth. MSC-derived EVs offer a multifaceted therapeutic strategy for corneal nerve regeneration by modulating both neuroinflammatory responses and regenerative signaling pathways. As seen in peripheral nerve injury, MSC-EVs can modulate this neuroinflammatory cascade by promoting macrophages toward an anti-inflammatory M2 phenotype, downregulating the production of proinflammatory cytokines, such as IL-1β, IL-6, and TNF-α, and reducing the expression of dendritic cell markers [20,31]. This immunomodulatory function contributes significantly to creating a permissive microenvironment for axonal regeneration in the cornea.

### 2.3. Stromal Regeneration

The stromal layer is the thickest layer of the cornea, consisting of 80% water, collagen fibers (types I, IV, V), proteoglycans and glycosaminoglycans, keratocytes (corneal fibroblasts), and a dense nerve network. The uniform arrangement of collagen and homeostatic function of keratocytes are essential for maintaining corneal transparency [34]. Stroma can partially regenerate after minor superficial injuries without basement membrane/Bowman’s layer disruption, with keratocytes repairing the ECM and restoring some transparency. Severe deep injuries with damage to Bowman’s layer often lead to fibrosis and scar formation due to myofibroblast differentiation and disorganized ECM deposition, resulting in corneal scar and haze [35].

Current treatments for corneal stromal pathologies, such as corneal transplantation and keratoprosthesis, have several limitations, such as immune rejection, corneal scarring, and incomplete regeneration [34,36]. A promising alternative involves activating the intrinsic regenerative potential of stromal cells or using stem cell-derived EVs to restore stromal structure and function [36,37]. Stem cell-derived extracellular vesicles exert regenerative effects on the corneal stroma, with key roles in ECM remodeling, inflammation suppression, and fibrosis inhibition.

**ECM Remodeling and Stromal Regeneration:** Stromal regeneration is driven by distinct stromal cell populations and their secretomes, particularly EVs. Studies showed that conditioned media from stromal fibroblasts (SFs) significantly enhance neurite outgrowth, highlighting SFs’ neuroregulatory role. SF secretions contain diverse proteins, including 33 unique neuroregulatory factors, and are enriched in pathways related to neurogenesis, wound healing, and matrix remodeling. SFs also express high mRNA expression of matrix remodeling enzymes (MMP2), tissue inhibitors of metalloproteinases (TIMP1), chemokines (MCP1), neuropeptides (substance P), and ECM components. Conversely, corneal stromal keratocytes (CSKs) express higher levels of ECM maintenance genes such as lumican and keratocan and IL-4. These findings suggest that SF-derived EVs support stromal regeneration by modulating neurogenesis, extracellular matrix remodeling, and inflammatory pathways, whereas CSKs contribute to stromal homeostasis and structural integrity. This underscores the importance of cell-type-specific EV profiles in corneal stromal regeneration [38].

Adipose-derived stem cell (ADSC)-derived exosomes promote corneal stromal regeneration by enhancing stromal cell proliferation, reducing apoptosis, and orchestrating ECM remodeling. Shen et al. showed that exosome treatment downregulates MMPs and upregulates ECM proteins like collagens and fibronectin, promoting matrix synthesis and stability. Exosomal protein and mRNA cargo induce an increase in ALDH expression after exosome treatment, pointing to a reversion of activated corneal stem cells (CSCs) toward a more quiescent, native phenotype, supporting tissue transparency and healing [39].

**Anti-inflammatory and Antifibrotic Effects:** EVs have demonstrated immunomodulatory and anti-fibrotic activity in deep corneal injuries. EVs derived from corneal stromal stem cells (CSSCs) promote stromal regeneration by modulating inflammation and fibrosis through their RNA and protein cargo. CSSC-EVs prevent corneal scarring by suppressing the expression of fibrotic markers such as Col3a1 and Acta2, as well as early pro-inflammatory genes, including Chi3l1, Lcn2, and Cxcl5, and reducing neutrophil infiltration [37]. Moreover, Yam et al. showed regenerative EVs from “healing” CSSC batches characterized by high levels of miR-29a and miR-381-5p significantly reduced corneal scarring and inflammation in vivo. These miRNAs downregulated key pro-fibrotic and inflammatory genes. Proteomic and transcriptomic analyses linked their targets to pathways involved in fibrosis suppression, immune modulation, and tissue regeneration. Notably, elevated miR-29a within CSSC-EVs strongly correlated with improved therapeutic efficacy, highlighting the promise of miRNA-enriched EVs as targeted therapies for corneal stromal regeneration and fibrosis mitigation [40].

Ong et al. illustrated that exosomes derived from mesenchymal stem cells (MSC-EVs) facilitate stromal regeneration through immunomodulation and fibrosis suppression. Upon topical application, MSC-EVs are rapidly taken up by corneal fibroblasts and myofibroblasts, where they reduce secretion of pro-inflammatory cytokines (IL-8, MCP-1, CXCL1) and promote macrophage polarization toward an anti-inflammatory M2 phenotype. At the molecular level, MSC-EVs treatment enhances epithelial wound closure and reduces stromal haze, fibrotic marker expression (fibronectin, collagen III, α-SMA), and neovascularization. Transcriptomic data show increased expression of anti-inflammatory genes (CD163, CD206, ARG1) and downregulation of pro-inflammatory genes (CD80, CD86, NOS2), indicating reprogramming of the wound environment. These findings further support the therapeutic potential of MSC-EVs in corneal stromal regeneration through coordinated immunomodulation and suppression of fibrosis [41].

Post-surgical stromal healing is tightly regulated by inflammatory and fibrotic signals and regenerative cues, with dysregulation leading to corneal haze. Kumar et al. showed that after PRK, the pro-fibrotic gene PREX1, upregulated in haze-prone corneas, enhances ECM production and fibrosis, especially under TGFβ stimulation. Conversely, knockdown of PREX1 reduces fibrotic gene expression but delays healing, highlighting its dual role. EVs engineered to suppress pro-fibrotic mediators like PREX1 or deliver anti-fibrotic regulators such as WNT3A and SOX17 offer a promising strategy to modulate aberrant stromal healing. These findings underscore the potential of EV-based therapies to restore corneal clarity after corneal surgeries [42].

### 2.4. Other EV Sources

Beyond MSCs, other EV sources also contribute to corneal regeneration.

Peripheral blood- and cord blood-derived EVs carry angiogenic regulators such as VEGF, PDGF, and angiopoietins, along with inhibitors like thrombospondin-1 (TSP1), allowing for a context-dependent vascular modulation [43].

Platelet-rich plasma (PRP) and platelet lysate EVs (PEVs) are abundant in growth factors (VEGF, PDGF, TGF-β, PF4) and adhesion proteins, supporting epithelial healing and stromal remodeling. However, their strong pro-angiogenic activity may limit use where corneal avascularity must be preserved [44].

CD34+ progenitor cell-derived EVs promote angiogenesis, mobilize endothelial progenitors, and accelerate wound healing in ischemic models [45].

Compared to MSC-EVs, these EV sources tend to be more angiogenic but less immunomodulatory, suggesting they may complement rather than replace MSC-EVs in corneal therapy [46].

## 3. Proteomic Profiling of EVs

Understanding the therapeutic potential of EVs in corneal regenerative medicine depends greatly on unraveling their complex protein cargo. Proteins carried within EVs are thought to be responsible for many of their regenerative effects, including modulation of inflammation, enhancement of epithelial wound healing, and stimulation of extracellular matrix (ECM) remodeling. To fully characterize these bioactive components, advanced proteomic profiling techniques have become indispensable.

### 3.1. EV Isolation Methods

EVs can be derived from a variety of biological sources, ranging from plants and microorganisms to mammalian cells and human biofluids such as blood, urine, saliva, and cerebrospinal fluid. In corneal research, EVs are typically isolated from mesenchymal stem cell (MSC) culture supernatants. Regardless of origin, the presence of contaminants such as albumin (from serum), Tamm–Horsfall protein (in urine), or lipoproteins (in plasma) can compromise downstream proteomic results. Therefore, choosing an appropriate isolation method is a foundational step in EV research [47].

**Magnetic Levitation-Based EV Isolation:** A novel approach for EV isolation, termed EV-Lev, employs non-magnetic polymer beads in a magnetic levitation system to enable selective capture and sorting of EV subpopulations. Unlike traditional immunomagnetic separation methods, which often suffer from bead aggregation and limited selectivity, EV-Lev uses beads that respond to magnetic fields based on their densities. These beads do not aggregate and are sorted along flow channels, allowing for individual beads with captured EVs to be visualized and characterized, akin to flow cytometry in microfluidic channels [48].

**Ultracentrifugation (UC):** UC, particularly its differential ultracentrifugation (dUC) and density gradient ultracentrifugation (DGUC) variants, remains one of the most common and historically important techniques for isolating EVs. These approaches rely on the separation of particles based on differences in size and density, making them effective for extracting extracellular vesicles from complex biological samples. Differential ultracentrifugation was the first widely adopted method in extracellular vesicle research and continues to be used as a mainstay in many laboratories due to its relatively simple protocol and easy availability [49].

In DGUC, samples containing extracellular vesicles are layered on density gradients (usually sucrose or iodixanol), which allows for more precise separation of extracellular vesicle subtypes while helping to reduce contamination from proteins and lipoproteins [50]. Although these methods are still considered reference standards for obtaining highly pure EVs, they have practical limitations: they are labor-intensive and require sophisticated ultracentrifugation equipment and technical expertise to perform properly [51]. Another challenge is that prolonged exposure to very high centrifugal forces (~100,000× *g*) can cause EVs to aggregate or undergo membrane damage, which may interfere with their biological function or downstream analyses [52]. In addition, factors such as rotor type, centrifugal force, and sample viscosity can affect EV recovery and purity, contributing to variability in studies and limiting reproducibility, especially in translational settings [53].

**Size exclusion chromatography (SEC):** SEC is increasingly favored for isolating EVs, particularly because it offers a gentle approach that better preserves the native structure and biological activity of EVs compared to conventional methods like ultracentrifugation. This technique works by sorting particles based on their size using a porous resin, commonly Sepharose CL-2B, allowing larger EVs to elute before smaller proteins and soluble contaminants. Since Böing et al. reintroduced SEC for EV purification in 2014, its ability to yield high-purity and functionally intact EVs has been supported by multiple studies [54].

Research comparing SEC with differential ultracentrifugation (dUC) and sedimentation-based methods has shown that SEC is more effective at minimizing protein carryover while maintaining EV-specific markers like CD9, CD63, and CD81 [52]. Notably, EVs derived from mesenchymal stem cells and isolated via SEC retained their immunosuppressive capabilities in T-cell assays, whereas this function was diminished in dUC-isolated counterparts. Additionally, the SEC has proven valuable in biomarker discovery from biological fluids such as plasma and urine, where it has helped identify distinct proteomic signatures in disease models like cardiomyopathy [55].

While SEC is generally easy to implement and adaptable to various lab environments, it often requires sample pre-processing such as centrifugation or ultrafiltration to reduce viscosity and adjust sample volumes. Although the upfront setup and manual handling may pose challenges, advances in SEC automation and its combination with other scalable methods (e.g., tangential flow filtration or affinity chromatography) are expanding their role in clinical-grade EV production [47].

**Tangential flow filtration (TFF):** TFF is rapidly gaining attention as a reliable, scalable, and GMP-friendly technique for EV isolation, particularly in clinical settings where purity and consistency are essential. Compared to traditional ultracentrifugation, TFF enables faster and gentler processing of large biofluid volumes while better preserving EV integrity. This is achieved by directing the sample to flow tangentially across a semi-permeable membrane, reducing membrane clogging and minimizing shear stress. The result is an efficient, continuous size and weight-based separation of vesicles [56].

Interestingly, TFF is considered a subset of a broader, innovative separation strategy known as Field-Flow Fractionation (FFF). Unlike methods that rely on direct particle binding or harsh processing, FFF technologies operate without a stationary phase, maintaining the native properties of EVs. These systems separate particles within a narrow laminar flow channel, guided by an externally applied field (or gradient) perpendicular to the flow direction. Depending on the type of force applied, thermal (thermal FFF), centrifugal (sedimentation FFF), electrical (electrical FFF), or cross/tangential flow (as in asymmetric flow FFF or AF4 and TFF/FFFF), EVs can be sorted based on their size, density, or surface charge [23].

Among these, AF4 has shown great promise in EV research due to its capacity for high-resolution fractionation, distinguishing vesicles with size differences as small as 10 nm. Key operating parameters, such as membrane pore size, channel thickness, and flow velocity, can be precisely adjusted to optimize separation. When integrated with analytical tools like UV absorbance (260/280 nm), multi-angle light scattering (MALS), or dynamic light scattering (DLS), AF4 enables real-time characterization of EV size, morphology, and purity [57].

Additionally, electrical FFF takes advantage of the mildly negative surface charge of EVs (commonly around 16 mV in PBS) to achieve charge-based separation. This is particularly effective in low-ionic-strength environments and provides a non-destructive, label-free approach to sorting vesicle populations [58].

Although FFF technologies are still in the early stages of widespread use in EV isolation, their ability to offer gentle, reproducible, and high-resolution separation without chemical modification or antibody binding makes them a compelling option for producing clinical-grade EVs in their native state [47].

**Charge-based isolation methods:** Charge-based separation methods take advantage of the natural negative charge found on the surface of EVs, which comes from certain membrane lipids like phosphatidylserine. A common technique uses magnetic beads with positive charges that attract and capture these negatively charged EVs without needing to target specific surface markers. These beads bind to EVs under carefully controlled conditions, like pH and salt concentration [59].

This method offers several benefits over traditional isolation techniques. It can deliver EVs with high purity, reduce unwanted protein contamination, and handle large volumes of biological fluids. Because of these features, it is well-suited for scalable processes that meet the strict standards required in research and clinical manufacturing [60].

For example, studies like Kozhevnikova et al. [61] have shown that magnetic bead-based separation efficiently isolates EVs from plasma while preserving important RNA and proteins, helping researchers find new biomarkers. Similarly, Ma et al. [62] used this approach to isolate EVs from mesenchymal stem cell cultures, achieving better yield and maintaining vesicle quality compared to traditional ultracentrifugation.

While this technique shows a lot of promise, it requires fine-tuning of buffer conditions to prevent unwanted binding and protect EV integrity during recovery. Still, its label-free, scalable, and automatable nature is attracting growing interest in industrial-scale production and therapeutic use of EVs [61].

**Immunoaffinity capture techniques:** This method, targeting EV surface markers (e.g., CD63, CD9, CD81), enables selective enrichment of specific EV subpopulations. Although this approach delivers highly pure vesicles, it typically yields a lower total amount and is cost-intensive [63]. This method works well for small samples and can be combined with magnetic beads to pull EVs out for further analysis or functional studies. But because no universal marker is found on all EVs, this technique only isolates specific populations of EVs, so a complete picture of all the EVs present is not obtained [64].

To capture a wider range of EVs, other binding agents have been developed, such as heparin or peptides that bind to heat shock proteins or specific lipids on the surface of EVs. Some approaches even target unique markers of EVs from specific tissues or cell types, which is particularly exciting for studying diseases such as cancer or heart disease. Although the immuno-affinity method provides highly purified samples of EVs, it typically yields less material and can be very expensive. For this reason, it is not yet widely used in routine clinical work, but ongoing developments could make it a more accessible and powerful tool for EV research and diagnosis [29,65].

**Microfluidic platforms:** Microfluidic platforms represent a significant innovation in EV isolation. These systems integrate size-based filtration, immunoaffinity, or electric field-based separation into compact chips, allowing for rapid, low-volume, and high-yield EV isolation. Examples include Exodisc and ExoTIC, which enable efficient EV isolation from various biofluids and support direct protein analysis on-chip via ELISA. Furthermore, dielectrophoresis-based and optically induced platforms have been used to separate EV subpopulations with high purity [66].

Despite rapid advances, isolating EVs with both high yield and high purity while maintaining their integrity and function remains a challenge, especially at the clinical scale. To address this, harmonized protocols for sample collection, handling, and characterization are essential. Interlaboratory validation using standardized workflows will also be critical for ensuring reproducibility in EV-based proteomics [67].

### 3.2. Analytical Platforms for EV Proteome Characterization

Once EVs are isolated, the next critical step is understanding what they carry, especially their protein cargo. These proteins can offer important clues about the EVs’ roles in health and disease, their therapeutic potential, and their value as biomarkers. To achieve this, researchers rely on a variety of analytical tools, each bringing their own strengths to the table.

**Mass Spectrometry (MS):** Mass spectrometry, especially liquid chromatography–tandem mass spectrometry (LC-MS/MS), is widely regarded as the gold standard for EV proteomic analysis. It allows scientists to identify and quantify a broad spectrum of proteins with high sensitivity, even those present in low amounts. Thanks to MS, we now have a clearer picture of how EV protein profiles differ by cell type and disease state, which is essential for discovering new diagnostic markers and therapeutic targets in fields like ophthalmology, oncology, and neurology [68]. To prepare EVs for MS analysis, researchers typically use detergent-free lysis methods, followed by enzymatic digestion of proteins into peptides. These peptides are then purified before entering the mass spectrometer. While MS is incredibly powerful, it requires expensive equipment, skilled personnel, and relatively large sample volumes, making it less accessible for some clinical settings [69].

**Western Blotting:** Western blotting is a classic technique still widely used in EV research. It helps verify whether key EV markers like CD9, CD63, CD81, and TSG101 are present, essentially confirming the success of the isolation process. Although it does not offer the depth or scale of MS, Western blotting is reliable for validating results and checking for the presence of specific disease-related or therapeutic proteins [70].

**ELISA and Bead-Based Assays:** Enzyme-linked immunosorbent assays (ELISAs) and multiplex bead-based systems (such as Luminex) are popular for their ability to detect and quantify specific EV proteins quickly and accurately. These essays are especially useful in clinical studies where large numbers of samples need to be analyzed. However, because they rely on antibodies, they are limited to detecting known proteins and require high-quality reagents to ensure accuracy [71].

**Nanoflow Cytometry:** Thanks to recent advances, flow cytometry has evolved to detect even nanoscale particles like EVs. Nanoflow cytometry enables researchers to analyze surface protein markers on individual EVs, revealing insights into subpopulations and heterogeneity that bulk methods might miss. While this is a promising tool, it still faces challenges related to sensitivity, fluorescent labeling, and data standardization [72].

**Microfluidic and Immunoaffinity-Based Technologies:** Newer lab-on-a-chip platforms are making it possible to isolate and analyze EV proteins in a single, miniaturized device. These microfluidic systems often use immunoaffinity capture or electric fields to sort and analyze EVs directly from small fluid samples. This makes them ideal for point-of-care diagnostics. However, many of these technologies are still in the experimental phase and may not yet be ready for widespread clinical use [73].

**Other Complementary Techniques:** Additional tools are also emerging, including capillary electrophoresis-based proteomics, protein microarrays, and even Raman spectroscopy. These methods are especially valuable when sample amounts are limited or when rapid analysis is needed, offering flexibility in different research or clinical contexts [74].

## 4. Proteome Variation

### 4.1. Priming and Preconditioning

MSC preconditioning can enhance the quality of MSC-exosomes [75]. These strategies are designed to modulate the cellular environment or apply physical stimuli to induce targeted alterations in the miRNA cargo of EVs, thereby enhancing their regenerative and immunomodulatory properties. Preconditioning MSCs with a variety of strategies, including hypoxia, cytokines, 3-dimensional culture, or oxidative stress, can significantly affect and reshape their proteomic profiles, enhancing their therapeutic efficacy in corneal disease models. Importantly, these preconditioning methods exert their effects primarily by altering the composition and functional capacity of the EVs released from MSCs, thereby ensuring that therapeutic benefits are conveyed in a cell-free format.

**Hypoxia:** Hypoxia preconditioning is a widely used strategy for priming MSCs and their derived exosome. In this method, the oxygen exposure is reduced, which enhances the proliferative capacity and genetic stability of MSCs [76]. Exposure of MSCs to hypoxic conditions can upregulate the expression of immunosuppressive factors such as prostaglandin E2, indoleamine 2,3-dioxygenase, and TGF-β, thereby enhancing the capacity to suppress T-cell proliferation and activation [77]. A previous study on exosomal miR-126 derived from UC-MSCs demonstrated that activation of HIF-1α under 1% oxygen conditions upregulated miR-126 levels in exosomes, subsequently promoting endothelial cell proliferation, angiogenesis, and migration [78]. These findings highlight that hypoxia-induced benefits of MSCs are largely mediated through the enriched cargo of their EVs.

**Inflammation:** Another pretreatment approach, known as cytokine preconditioning, involves stimulation with cytokines and inflammatory factors. This approach has been demonstrated to increase paracrine signaling and improve the therapeutic efficacy of MSC-derived exosomes. Stimulation of MSCs with inflammatory cytokines like interferon-gamma (IFN-γ) and tumor necrosis factor-alpha (TNF-α) has been shown to upregulate the expression of key immunomodulatory molecules such as programmed death-ligand 1 (PD-L1) and induce the secretion of anti-inflammatory cytokines, including interleukin-10 (IL-10) [79]. Exposure to TNF-α enhances the release of exosomes with stronger anti-inflammatory properties, notably enriched with inflammation-suppressing microRNAs such as miR-146a [80,81]. Notably, exposure to IFN-γ has been shown to enhance MSC functionality by modifying cell morphology [82] or promoting the upregulation of immunosuppressive mediators, including IDO, PGE2, PD-L1, and TSG-6 [83]. These immunomodulatory effects are transmitted to recipient cells primarily through the EVs secreted by MSCs, emphasizing the critical role of EV cargo in mediating therapeutic outcomes.

**LPS:** Lipopolysaccharide (LPS) is commonly used to precondition MSCs due to its strong capacity to activate immune responses. Interestingly, low-dose LPS has been shown to exert protective effects in various disease models. Different concentrations of LPS stimulate MSCs to release exosomes with distinct functional properties, likely influenced by dose-dependent variations in their miRNA content [84]. A study by Lohan et al. [85] demonstrated that MSCs, particularly when preconditioned with LPS and IFNγ, exhibit enhanced immunoregulatory effects that significantly prolong corneal allograft survival in a high-risk transplantation model. This indicates that the therapeutic impact of LPS-primed MSCs is largely EV-mediated.

**Dimensional (3D) Culture:** Culturing MSCs in a three-dimensional (3D) environment represents a form of preconditioning designed to replicate the physiological conditions encountered in vivo. Specifically, 3D culture can enhance the secretion of factors associated with cell survival, proliferation, and vascularization [86,87]. Another study investigates how 2D versus 3D culture conditions influence the composition and therapeutic efficacy of the MSC secretome. In vitro and ex vivo corneal wound healing models were used to assess regenerative outcomes. Results reveal that 3D culture enhances secretion of pro-regenerative factors, leading to improved epithelial repair and anti-inflammatory effects [88]. These enhancements are mediated by EVs, which carry the bioactive proteins and miRNAs responsible for the observed therapeutic benefits.

**Serum-free Media:** Serum-free culture of MSCs has emerged as a valuable strategy due to its influence on cellular functions and the composition of secreted exosomes. These modifications improve the therapeutic efficacy of MSC-EVs, highlighting serum-free culture conditions as a promising approach for applications in regenerative medicine. Serum-free culture media have been shown to modulate mitochondrial antioxidant activity and alter the metabolic profile [89]. In a previous BMSC study, serum-free culture conditions led to an increased abundance of extracellular vesicle-associated miR-17-92 and accelerated cell proliferation and migration (REF) [90]. This underscores that serum-free conditions enhance the regenerative potential of MSCs primarily by optimizing the composition and function of the EVs they release.

**Oxidative stress:** Oxidative and sulfide compounds can influence the biological activity of MSCs by altering miRNA profiles through the modulation of miRNA expression and packaging within EVs. As a result, these compounds can enhance the anti-inflammatory, antioxidative, and cytoprotective properties of the vesicles.

### 4.2. Source-Dependent

While MSCs remain the most extensively studied source of regenerative EVs in corneal repair, it is important to note that other cell types also secrete EVs with therapeutic potential. Corneal epithelial cells release EVs containing structural proteins and ECM regulators that enhance epithelial barrier function and wound closure. Endothelial cell-derived EVs contribute to stromal remodeling and vascular homeostasis, whereas cord-lining stem cells and platelet-derived EVs carry bioactive proteins and growth factors that support angiogenesis, immune modulation, and tissue regeneration. These diverse EV sources highlight the influence of cellular origin on EV cargo composition and underscore the need for comparative studies to identify optimal EV populations for corneal therapy [91].

MSCs used in ocular disease treatment are primarily derived from the umbilical cord, bone marrow, adipose tissue, and corneal stroma. As shown in Table 1 and Table 2 hese sources possess inherent biological differences that may substantially influence the therapeutic properties of their EVs [92]. The distinct miRNA profiles of MSC-derived exosomes reflect both the tissue origin and physiological state of the donor cells, with exosomes from adipose tissue, umbilical cord, and bone marrow MSCs each carrying a unique set of miRNA species [76,93]. Therefore, the regenerative and immunomodulatory effects observed with different MSC sources are largely mediated through their EVs rather than the cells themselves. Nevertheless, the best source of MSCs has not yet been identified. Previous studies have undertaken comparative analyses of the molecular and functional characteristics of MSCs derived from different tissue sources, employing independent omics-based approaches. These include transcriptional profiling [94,95], proteomic analyses, and secretome profiling [96,97].

**Umbilical cord MSC:** UC-MSCs are immunologically less mature than adult stem cells, which enables them to better tolerate HLA mismatches and elicit a diminished immune response in allogeneic recipients [112]. Liu et al. demonstrated that UC-MSCs can adopt a keratocyte-like phenotype, promote collagen matrix organization, and enhance corneal transparency in lumican-null mice [113]. Moreover, they can accelerate corneal epithelial growth and improve corneal wound healing [114]. Ma et.al examined the therapeutic effect of UM-MSc on the corneal wound healing. Treatment with UM-MSC enhanced cell cycle progression in cornea cells through the upregulation of key proteins, proliferating cell nuclear antigen, Cyclin A, Cyclin E, and CDK2. Additionally, inflammation was reduced by suppressing cytokines such as TNF-α, IL-1β, IL-6, and CXCL-2. Furthermore, apoptosis was effectively suppressed by reducing the levels of pro-apoptotic markers, including Bax and cleaved Caspase-3, and increasing Bcl-2.(REF) [101]. The antiangiogenic and regenerative effects of corneal MSCs are largely conveyed via their secreted EVs, which are enriched in sFLT-1, PEDF, and other bioactive factors that modulate endothelial and stromal cell behavior. Another study that compared the angiogenic potential of secretomes derived from Wharton’s jelly UC-MSCs, AD-MSC, and BM-MSC, found that Wharton’s jelly UC-MSCs secretome exhibited a more comprehensive angiogenic protein profile, characterized by higher concentrations of angiogenesis-related factors, followed by the BM-MSC secretome. In contrast, AD-MSC secretome lacked key angiogenic proteins and exhibited significantly lower expression levels of most detected proteins compared to the other sources (REF) [115].

**BM-MSC:** Previous study showed that BMSC-EVs target the damaged cornea, modulating cell death, inflammation, and angiogenic pathways in the injured tissue, thereby promoting faster recovery of corneal damage [16]. Pomatto et al. [103] demonstrated that BMSC-EVs contain proteins involved in inflammation, cell adhesion, fibrosis, and apoptosis, such as IL13, IL1RL2, IL5, IL10, and FN1. In another study, mild, self-limiting conjunctival inflammation was observed at the injection site in rabbit eyes receiving human MSCs, but not in those injected with rabbit MSCs. Subconjunctival administration of MSCs is safe and effective in promoting corneal epithelial wound healing in animal models. Zhou et al. [99] (REF) demonstrated that BMSC-Exos significantly promoted the proliferation and migration of human corneal epithelial cells (HCECs) in a dose-dependent manner. This effect was primarily mediated through activation of the p44/42 MAPK signaling pathway, as evidenced by partial inhibition with the MAPK pathway inhibitor U0126. In vivo, administration of BMSC-Exos led to a marked reduction in corneal inflammation, fibrosis as indicated by decreased expression of α-smooth muscle actin (α-SMA) and neovascularization, reflected by reduced CD31 levels. These findings underscore the therapeutic potential of BMSC-Exos in promoting corneal repair and regeneration. Carter et al. [88] examined the impact of paracrine factors from BMSCs on corneal fibroblasts. Among the study groups, MSCs co-cultured with electrospun fibers demonstrated a significantly faster wound closure rate in vitro compared to other conditions. In the ex vivo model, both MSCs alone and the MSC-electrospun fiber co-culture demonstrated comparable wound closure rates and significantly reduced corneal opacity, outperforming the control group. Notably, the MSC-electrospun fiber co-culture also resulted in higher cell viability, suggesting enhanced regenerative potential.

**AD-MSCs:** Transplanted AD-MSCs decreased ocular surface inflammation, inhibited corneal neovascularization, and partially restored limbal and corneal epithelial phenotypes [104]. Adipose tissue fragments used for MSC isolation may contain a heterogeneous mixture of preadipocytes, mesenchymal stem cells, endothelial progenitor cells, mast cells, lymphocytes, and macrophages, which may contribute to the accelerated differentiation into myofibroblast-like cells [105]. Nevertheless, Galindo et al. [104] demonstrated that AD-MSCs, when cultured on an amniotic membrane and transplanted onto the ocular surface, promote ocular surface regeneration in a rabbit model of limbal stem cell deficiency. A comparative study of protein synthesis and secretion of MSCs derived from bone marrow, adipose tissue, and Wharton’s jelly (WJ) demonstrated notable differences in their biological profiles [106]. WJ-MSCs exhibited the highest proliferative capacity, followed by AD-MSCs. Surface marker expression differed in CD54 and CD146. WJ-MSCs secreted higher levels of chemokines, pro-inflammatory cytokines, and growth factors, while AD-MSCs exhibited a stronger pro-angiogenic profile and released greater amounts of extracellular matrix components and matrix metalloproteinases. Shibata et.al showed that AD-MSC can suppress epithelial–mesenchymal transition-related gene expression, which plays a role in the loss of cell–cell interactions in the cornea, and also inhibits TGF-β [107]. In another study, comparative proteomic analysis revealed that the secretomes of MSCs derived from sources such as the placenta and Wharton’s jelly exhibit greater compositional diversity than those of AD-MSCs and BM-MSCs [108].

**Cornea MSC:** Proteome array analysis revealed that preconditioned corneal MSCs increase expression of angiogenesis inhibitors, making them well-suited for treating corneal neovascularization [109]. Corneal stromal stem cells can differentiate into keratocyte-like cells and deposit native stroma-like extracellular matrix when cultured in serum-free conditions supplemented with bFGF and TGFβ3, demonstrating their potential for stromal regeneration [110]. The antiangiogenic and regenerative effects of corneal MSCs are largely conveyed via their secreted EVs, which are enriched in sFLT-1, PEDF, and other bioactive factors that modulate endothelial and stromal cell behavior. A previous study demonstrated that corneal MSC secretome attributed to an increase in high levels of antiangiogenic factors, including sFLT-1 and PEDF, and decreased expression of antiangiogenic factors like VEGF-A. In vivo, corneal MSCs prevented corneal neovascularization after injury [111].

### 4.3. Protein and microRNA Cargo

Extracellular vesicles (EVs) carry a rich cargo of both proteins and microRNAs (miRNAs), which work together to support corneal repair and regeneration. While the protein components of EVs provide direct structural and signaling support, miRNAs fine-tune gene expression in recipient cells, amplifying the regenerative potential. Several studies have identified key miRNAs in MSC- and corneal cell–derived EVs that influence multiple aspects of corneal healing. For epithelial repair, EVs enriched in miR-21, miR-27a, and miR-146a accelerated wound closure by modulating the PI3K/Akt and NF-κB pathways. Antifibrotic effects are mediated by miR-29 and miR-133b, which suppress collagen type III and α-SMA expression, thereby reducing stromal scarring [116].

EVs carrying miR-182 and miR-183 promote nerve regeneration by enhancing axonal elongation and sensory recovery through cytoskeletal regulation. In addition, angiogenesis is tightly controlled by a balance of miRNAs: miR-132 and miR-210 enhance vascular sprouting, whereas miR-200b and miR-16 inhibit pathological angiogenesis. EVs also contribute to immune regulation, with miR-21 and miR-181a downregulating pro-inflammatory mediators, such as IL-6 and TNF-α, while promoting anti-inflammatory signaling [117].

Together, these proteins and miRNAs act in concert to orchestrate a multifaceted regenerative response in the cornea. Understanding the interplay between proteomic and miRNA cargo is essential for fully appreciating the therapeutic potential of EVs, and integrating proteomic with transcriptomic analyses will be critical for optimizing EV-based strategies for corneal repair [118].

## 5. Functional Roles of Identified Proteins

EVs derived from mesenchymal stem cells (MSCs) exert their regenerative effects largely through the diverse array of bioactive proteins they carry. Proteomic profiling has revealed that these vesicles contain critical mediators of corneal repair, including proteins involved in angiogenesis modulation, anti-fibrotic activity, and neuroprotection. These proteins regulate inflammation, promote epithelial and stromal regeneration, and support nerve repair, highlighting the potential of EVs as a cell-free therapeutic modality in corneal regenerative medicine [14].

### 5.1. Proteins Involved in Angiogenesis and Vascular Modulation

The cornea’s avascular nature is essential for optical transparency, yet EV-mediated angiogenic regulation demonstrates context-dependent therapeutic plasticity. Platelet-derived EVs (PEVs) contain pro-angiogenic mediators, including VEGF-A, MMP-2, and MMP-9, while MSC-derived EVs deliver coordinated angiogenic signals through VEGF, FGF, and PDGF expressions [119]. Pro-inflammatory cytokines IL-6 and IL-8 within MSC-exosomal cargo further amplify angiogenic responses by promoting endothelial cell migration and proliferation [120].

EVs derived from MSCs contain both pro-angiogenic factors and anti-angiogenic molecules. The balance of these factors is influenced by the source of MSCs and environmental conditions such as hypoxia or immune activation [121]. For example, hypoxia-preconditioned MSCs produce EVs enriched in VEGF and angiopoietin-1, which enhance epithelial healing in ischemic corneal wounds via activation of PI3K/AKT and ERK1/2 signaling pathways. Conversely, EVs cultured under normoxia may favor an anti-angiogenic profile by upregulating TSP-1 and PEDF, which inhibit endothelial cell proliferation and induce apoptosis [122].

This dual regulatory capacity illustrates the source- and context-dependent flexibility of MSC-EVs. In preclinical models, MSC-EVs reduced corneal neovascularization, suppressed macrophage infiltration, and downregulated angiogenesis-associated genes such as VEGF, MMP-2, and MMP-9 [14]. Proteomic tuning of EVs may, thus, allow for targeted modulation of angiogenesis, promoting revascularization in ischemia while preserving corneal avascularity to maintain clarity.

Liu et al. [123] reported that platelet-derived extracellular vesicles (PEVs), despite carrying pro-angiogenic factors, exhibit primarily anti-angiogenic effects in corneal neovascularization models. This paradox is attributed to the intricate balance of signaling molecules, where anti-angiogenic mediators such as PF4, thrombospondin-1 (TSP-1), and endostatin act in concert with regulatory microRNAs (miR-22, miR-185) to maintain corneal avascularity during the healing process [123].

Saccu et al.’s [16] matrix metalloproteinase-14 (MMP-14) represents a critical regulatory node in corneal fibroblast-derived EV function. MMP-14 knockout studies reveal compensatory upregulation of pro-angiogenic proteins and reduced expression of angiogenesis regulators BMP1, SRPX2, and filamin-A. Bone marrow-derived MSC-EVs (BMSC-EVs) demonstrate superior anti-angiogenic efficacy through targeted TGF-β pathway suppression, downregulating expression of the growth factors Vegfa, Vegfd, and receptor tyrosine kinases expression of the VEGF receptors. Flt1, Kdr (Flk1), and Flt4 also followed a similar trend, increasing significantly only in the VHL-treated injured corneas on day 3 with respect to EV-treated ones [16].

Engineered EVs incorporating anti-angiogenic peptides such as KV11 have demonstrated enhanced therapeutic efficacy. Similarly, exosomes derived from lens epithelial cells enriched with miR-146a-5p offer targeted anti-angiogenic therapy. In addition, EVs from human placenta-derived mesenchymal stem cells (MSCs) significantly downregulate angiogenesis-related genes, including MMP2, MMP9, and VEGF-A, in injured corneal tissue [14].

Tao et al. [14] investigated the therapeutic potential of human placenta-derived mesenchymal stem cell (hP-MSC)-derived EVs for the treatment of corneal alkaline injuries. Their results showed that repeated topical application of hP-MSC-EVs increased the proliferation and migration of corneal epithelial cells, reduced apoptosis and inflammation, and improved epithelial repair both in vitro and in vivo. EVs also suppressed pathological angiogenesis and reduced proinflammatory cytokines (TNF-α, IL-1β, IL-8, NF-κB) while increasing IL-10, which resulted in reduced angiogenesis and improved tissue integrity. These findings suggest that hP-MSC-derived EVs offer a promising cell-free therapeutic strategy for corneal wound healing by modulating inflammation, angiogenesis, and epithelial remodeling [14].

### 5.2. Anti-Fibrotic and Extracellular Matrix Remodeling Proteins

Tissue fibrosis represents a major barrier to regenerative healing, particularly in the cornea, where stromal scarring impairs transparency and visual acuity. Fibrosis results from dysregulated remodeling of the extracellular matrix (ECM), characterized by excessive deposition of collagens and fibronectin, aberrant myofibroblast activation, and chronic inflammation [124].

MSC-derived EVs have demonstrated anti-fibrotic effects through several mechanisms. They modulate ECM turnover by delivering regulatory proteins such as decorin, MMP-14, TIMP-2, and TGF-β pathway inhibitors (e.g., SMAD7), which suppress fibrogenic signaling cascades and limit myofibroblast differentiation [125]. In murine models, corneal stromal stem cell-derived EVs reduced expression of fibrotic genes (e.g., Col3a1, Acta2), prevented neutrophil infiltration, and restored stromal clarity, showing effects comparable to live-cell therapies [125].

Young et al. [1] investigated how different layers of the cornea, particularly the epithelium and stroma, communicate via EVs during healing. They found that extracellular vesicles isolated from human corneal epithelial cells (HCEs), keratocytes (HCKs), fibroblasts (HCFs), and myofibroblasts (HCMs) were similar in size and charge, but their protein content varied significantly depending on the cell of origin. Myofibroblast extracellular vesicles (HCM-EVs) were rich in fibrosis-associated proteins such as COL6A1, COL6A2, MMP1, MMP2, TIMP1, and TIMP2, suggesting their role in scar formation. On the other hand, epithelial cell extracellular vesicles (HCE-EVs) carry proteins involved in stress response and protein synthesis regulation, such as RPS21, RALA, and EIF3H, which are linked to the EIF-2 signaling pathway. All types of extracellular vesicles expressed the common marker CD81 and lacked the Golgi protein GM130 [1].

Shojaati et al. revealed that EVs secreted by corneal stromal stem cells (CSSC) play a critical role in promoting corneal repair. In a mouse model of corneal injury, EVs significantly reduced stromal fibrosis, prevented neutrophil infiltration, and lowered the expression of fibrosis-related genes such as Col3a1 and Acta2. These regenerative effects were comparable to those of live CSSCs, while EVs from non-stem cell sources, like HEK293T cells, showed no such benefit. This study further showed that CSSC EVs deliver microRNAs (miRNAs) to corneal cells, and this function is dependent on Alix, a key protein involved in EV formation. Silencing Alix led to a sharp decline in EV-associated miRNAs and eliminated the therapeutic benefits, indicating that the regenerative potential of CSSCs relies on EV-mediated miRNA transfer [1,37]

### 5.3. Neuroprotective and Nerve Regeneration Factors

Corneal nerves are essential for maintaining ocular surface health through the regulation of epithelial integrity, tear secretion, and the blink reflex. Damage to corneal innervation, whether from trauma, surgery, or disease, can lead to neurotrophic keratopathy and delayed epithelial repair. The neuroprotective role of MSC-derived EVs has gained increasing attention due to their capacity to deliver proteins that support nerve regeneration and neuronal survival. Proteomic analysis of EVs has identified a range of neurotrophic factors, including nerve growth factor (NGF), brain-derived neurotrophic factor (BDNF), glial cell line-derived neurotrophic factor (GDNF), VEGF, and fibroblast growth factors (FGFs) [126,127]. These proteins facilitate axonal extension, stimulate synaptic repair, and enhance neurogenesis. NGF accelerates epithelial healing and reinnervation [128], while BDNF supports axonal regeneration and improves sensory recovery.

Beyond neurotrophins, EVs carry anti-apoptotic and antioxidant proteins such as Bcl-2, peroxiredoxins, and superoxide dismutase (SOD1), which help mitigate oxidative stress in injured nerves [129]. Heat shock proteins (e.g., HSP70, HSP90) further stabilize neuronal survival under stress conditions by preventing protein misfolding and aggregation [130].

The proteomic content of EVs varies by MSC source and microenvironment. Hypoxic preconditioning has been shown to enhance the expression of neuroprotective proteins, improving the regenerative capacity of EVs. In vivo studies have confirmed that topical or subconjunctival delivery of MSC-EVs enhances nerve density, restores corneal sensation, and promotes epithelial healing, correlating with their enriched neurotrophic protein content [131].

### 5.4. Anti-Inflammatory and Immunomodulatory Proteins

Ocular inflammation plays a central role in the pathogenesis of many vision-threatening diseases, including dry eye disease, corneal injury, and diabetic retinopathy. EVs, particularly those derived from mesenchymal stem cells (MSCs), offer a promising, cell-free therapeutic strategy by modulating immune responses and regulating key inflammatory pathways.

EVs exert their immunomodulatory effects by influencing immune cell phenotypes and inflammatory cytokine production. For example, EVs from bone marrow–derived MSCs (BMSC-EVs) have been shown to significantly attenuate inflammation following corneal alkali burn injury. They reduce levels of pro-inflammatory cytokines such as TNF-α, IL-1β, and IL-6, thereby supporting corneal healing while minimizing tissue damage [14]. Similarly, human placenta-derived MSC-EVs (hP-MSC-EVs) have demonstrated robust anti-inflammatory activity in both in vitro and in vivo models. These EVs suppress inflammatory cytokine production, reduce angiogenesis, and promote epithelial regeneration, largely through inhibition of the NF-κB signaling pathway and upregulation of IL-10 [14].

Ong et al. [41] investigated the therapeutic effects of mesenchymal stem cell-derived exosomes in a rat model of corneal scarring. The topical application of EVs for five days significantly improved corneal wound healing, with faster epithelial closure, reduced stromal opacity, and reduced fibrosis compared with the PBS-treated control group. EVs treatment also reduced corneal nervousness and modulated inflammation by shifting macrophage polarization toward a repair-promoting M2 phenotype (CD163+, CD206+) while decreasing proinflammatory cytokines (IL-1β, TNF-α, IL-8, CXCL1) and increasing IL-10 levels [41].

Chan et al. [132] investigated the effects of umbilical cord MSC-derived exosomes (UCMSC-EVs) in a rat model of severe dry eye disease (DED) induced by lacrimal gland removal. UCMSC-EV treatment led to a significant reduction in corneal neovascularization, epithelial disruption, and inflammation. The exosome-treated group demonstrated improved epithelial thickness, decreased fluorescein staining, and regulation of α-SMA expression and apoptosis. Additionally, expression of pro-inflammatory cytokines IL-1β and TNF-α was significantly reduced. These findings suggest that UCMSC-EVs support ocular surface healing and immune regulation, offering a promising non-cellular therapy for advanced DED [132].

Given their strong immunomodulatory potential, mesenchymal stem cells (MSCs) and their extracellular vesicles (MSC-EVs) have been incorporated into several clinical trials targeting NLRP3-driven ocular inflammation. A recent double-blind, randomized clinical trial in Denmark (NCT04615455) explored the efficacy of adipose tissue-derived MSCs (AT-MSCs) in treating severe aqueous-deficient dry eye disease (ADDE) due to Sjögren’s Syndrome. Patients received either AT-MSCs or a placebo via transconjunctival injection into the lacrimal gland, with outcomes such as tear stability, secretion, and ocular surface health assessed over four months. While completed in early 2023, results are still pending publication [133].

Another ongoing trial (NCT05147701) in Argentina, Antigua, and Barbuda is investigating intravenous and sub-tenon delivery of umbilical cord-derived MSCs (UC-MSCs) for various eye diseases, including monitoring for adverse events over a four-year period. In China, a study at Sun Yat-sen University (NCT04213248) is evaluating the topical application of umbilical MSC-derived exosomes in dry eye disease associated with chronic graft-versus-host disease (cGVHD). Patients receive exosome drops four times daily for two weeks, with assessments of ocular surface health continuing for 12 weeks. Results are expected by the end of 2023 [133].

### 5.5. Cell Survival and Proliferation Factors

Effective corneal healing requires coordinated cell survival, proliferation, and migration. EV-mediated delivery of survival and proliferation factors represents fundamental mechanisms underlying regenerative potential.

Desjardins et al. [134] demonstrated that exosomes derived from human corneal epithelial cells (hCECs), fibroblasts (hCFs), and endothelial cells (hCEnCs) play a pivotal role in coordinating corneal wound healing. Their in vitro findings showed that exosomes from all three corneal cell types significantly enhanced wound closure in scratch-injured hCECs. Mechanistically, these vesicles activate signaling pathways such as HSP27, STAT, β-catenin, GSK-3β, and p38, which are key regulators of cell proliferation and migration. Further examination of gene expression profiles revealed that the effects of exosomes are highly dependent, with each type of exosome altering a distinct set of genes in target cells. For example, in hCECs, exposure to exosomes leads to increased expression of genes associated with proliferation and migration, while downregulating genes associated with differentiation [134].

An et al. [23] investigated the therapeutic potential of bone marrow-derived mesenchymal stem/stromal cell (MSC-S) secretome, focusing on the role of extracellular EVs and exosomes in corneal epithelial wound healing. Their in vitro studies using human corneal epithelial cells (HCECs and HCLEs) showed that MSC-CM culture medium significantly increased cell proliferation, while removal of EVs from the culture medium reduced this effect. Both in vitro and in vivo experiments confirmed that wound healing was more effective with higher doses of MSC-S and that the presence of EVs was essential for optimal healing. They also found that the secretome collected after 72 h had a greater regenerative effect than the secretome collected at 48 h. Importantly, MSC-S maintained its wound healing capacity after one freeze–thaw cycle and remained stable for up to four weeks at 4 °C. These findings emphasize that extracellular vesicles/exosomes are key bioactive components of the MSC secretome [23].

Young et al. [10] investigated how extracellular vesicles from keratocytes, fibroblasts, and myofibroblasts affect corneal epithelial wound healing. They isolated and characterized these extracellular vesicles and found that they differentially affected the migration, proliferation, and apoptosis of human corneal epithelial cells in scratch assays. Compared with HCK-EVs, HCF, and HCM-EV extracellular vesicles expressed higher levels of surface markers such as CD63, CD81, ITGAV, and THBS1, although their physical properties were largely similar. Proteomic analysis revealed that HCM-EVs were significantly enriched in proteins associated with cell motility, particularly CXCL6, CXCL12, MMP1, and MMP2, compared with extracellular vesicles from the other two cell types. Functionally, treatment with HCM-EVs increased the migration, velocity, and proliferation of HCE cells more effectively than with HCK-EVs or HCF-EVs [10].

## 6. Challenges and Opportunities

Despite the promising therapeutic potential of mesenchymal stem cell-derived extracellular vesicles (MSC-EVs) in corneal regeneration, several key challenges must be addressed to enable their successful translation into clinical practice. One of the major obstacles in the field of EV therapeutics is the lack of standardized protocols for EV isolation, purification, and characterization. Variability in isolation techniques, ranging from ultracentrifugation to size-exclusion chromatography and commercial precipitation kits, can result in heterogeneous EV preparations with differences in purity, yield, and protein cargo [135]. This lack of consistency hampers the reproducibility of findings across studies and complicates regulatory assessment. Moreover, differences in source cell types, culture conditions, and preconditioning regimens (e.g., hypoxia, inflammatory stimuli) further influence EV content and therapeutic function [136]. Standardized criteria for defining MSC-EVs, such as the Minimal Information for Studies of Extracellular Vesicles guidelines, have been proposed but are not yet uniformly applied.

To advance clinical applications, we have expanded the discussion to include concrete details on clinical translation. Specifically, we now address the development of robust, scalable, and reproducible EV production pipelines with validated quality control metrics, including size distribution, sterility, and potency assays relevant to corneal biology. We also discuss challenges such as GMP-compliant manufacturing, standardization of EV preparations, and emerging bioengineering strategies for preconditioning or engineering EVs to enhance therapeutic efficacy [137]. Although pre-clinical studies demonstrate the regenerative and immunomodulatory efficacy of MSC-EVs in corneal models, clinical translation remains in its early stages. There are currently few registered clinical trials investigating EVs in ocular disease, and none specifically targeting corneal nerve or stromal regeneration [23].

Nonetheless, the inherent low immunogenicity, cargo modularity, and ability to cross biological barriers position MSC-derived EVs as a uniquely promising platform for cell-free regenerative therapies [138]. As proteomic technologies and bioengineering approaches evolve, there is a substantial opportunity to design next-generation EVs with enhanced targeting, controlled release, and customizable protein profiles tailored to the regenerative needs of corneal tissues.

### 6.1. Clinical Applications

While much of the research on EVs in ophthalmology is still preclinical, progress toward clinical translation is accelerating. Early-phase trials and regulatory discussions highlight both the therapeutic potential of EVs and the practical challenges in bringing them to patients.

Several clinical studies are exploring or have explored EV-based therapies in ocular conditions:

**Dry Eye Disease (DED):** A Phase I/II trial in China is testing the topical application of umbilical cord MSC-derived EVs (UC-MSC-EVs) in patients with graft-versus-host disease-associated DED. This study evaluates ocular surface integrity, tear film stability, and safety NCT04213248.

**Autoimmune-related DED:** A randomized trial in Denmark investigated adipose-derived MSC injections into the lacrimal gland for Sjögren’s syndrome. Although cell-based, this study provides insights into the potential for EV-based lacrimal therapies NCT04615455.

**Systemic EV therapies:** Broader clinical trials evaluating EVs for wound healing and inflammatory disorders may provide useful guidance for ophthalmic applications.

### 6.2. Challenges in Translation

Several hurdles must be overcome to advance EV-based therapies beyond the proof-of-concept stage:

**Standardization of Isolation and Characterization:** EVs isolated using different methods can vary widely in proteomic content, making reproducibility a challenge. The MISEV 2023 guidelines recommend thorough reporting of isolation procedures, purity, and cargo composition to facilitate comparisons across studies [139].

**Scalable Manufacturing:** Translating EVs to the clinic requires GMP-compliant, large-scale production. Techniques like tangential flow filtration (TFF), size-exclusion chromatography (SEC), and hybrid MagNet protocols are emerging as viable solutions [140].

**Potency and Quality Control:** Regulatory authorities require functional assays to confirm EV activity. For corneal applications, relevant readouts include inhibition of myofibroblast differentiation, promotion of epithelial wound closure, and modulation of angiogenesis. Standardization of these assays remains a challenge [141].

**Safety Considerations:** Although EVs are generally less immunogenic than cells, concerns remain about off-target effects, angiogenic potential (especially for blood- or platelet-derived EVs), and the possible transfer of oncogenic proteins or nucleic acids. Long-term safety monitoring in clinical trials is essential [142].

## 7. Summary and Outlook

Interest in EVs, particularly those derived from MSCs, is rapidly increasing, indicating a move toward more innovative, cell-free solutions for corneal repair. This review has shown how proteins carried in EVs play a pivotal role in processes such as reducing inflammation, repairing the epithelium, rebuilding the stromal tissue, and supporting nerve regeneration. Thanks to advanced tools such as LC-MS/MS, we now have a much clearer picture of how the protein content of EVs is shaped not only by the source cell type but also by surrounding conditions, such as hypoxia or inflammation. Notably, EVs from different MSC sources and even from different corneal cell types have unique protein “signatures” that indicate their specialized roles in healing or scarring. These differences point to the importance of accurately characterizing EVs to design targeted and effective therapies. By bringing together the latest findings on EV protein content, this review helps pave the way for the development of the next generation of personalized, cell-free therapies, providing new hope for patients in need of corneal repair, especially in the face of a shortage of donor tissue.

## Figures and Tables

**Table 1:** Comparative proteomic data of extracellular vesicles from different sources of mesenchymal stem cells (relevant to corneal regeneration).

MSC Source	Key Proteomic Features of EVs	Functional Implications	Ref.
Corneal Stromal MSCs (cMSCs)	- Express angiogenesis inhibitors (TSP1, PEDF) - Stromal proteins (lumican, keratocan) - Low levels of pro-angiogenic molecules	- Anti-angiogenic, immune-tolerant - Ideal for corneal clarity and transparency	[73]
Bone Marrow (BM-MSCs)	Proteins involved in apoptosis modulation (BAX, BCL2) - Inflammatory and fibrosis-related proteins (IL13, FN1, IL1RL2, IL5, IL10) - Extracellular matrix (ECM) remodeling proteins	- Reduces inflammation and fibrosis - Supports stromal healing and immune regulation	[98,99]
Adipose Tissue (AD-MSCs)	- High levels of ECM proteins and MMPs (MMP2, MMP9) - Pro-angiogenic factors (VEGF, ANGPTL4)	- Strong pro-angiogenic profile - Useful for wound healing, but may induce neovascularization Galindo	[100]
Umbilical Cord (UC-MSCs)	Enriched in growth factors (VEGF, HGF, IGF) - Anti-inflammatory proteins (TSG-6) - Stress response proteins	- Promotes epithelial wound healing - Enhances stromal regeneration	[101]
Wharton’s Jelly (WJ-MSCs)	- Enriched in chemokines, IL-6, pro-inflammatory cytokines - High growth factor and anti-apoptotic content	- High proliferation and paracrine signaling - Potential for nerve and stromal regeneration	[102]

**Table 2:** Key proteins and bioactive molecules identified in MSC-derived extracellular vesicles (EVs), categorized by source, functional role, and demonstrated effects in corneal models.

MSC Source	Key EV Proteins/miRNAs	Functional Role	Demonstrated Effect on Corneal Models	Reference
Umbilical Cord MSC (UC-MSC)	TSG-6, PD-L1, IL-10, miR-126	Anti-inflammatory, immunomodulatory, angiogenesis promotion	Reduced corneal inflammation, promoted endothelial proliferation, enhanced epithelial migration	[77]
Bone Marrow MSC (BM-MSC)	MMPs, TIMPs, FN1, IL-13, IL-1RL2	Anti-fibrotic, ECM remodeling, immunomodulatory	Reduced α-SMA expression, decreased neovascularization, enhanced epithelial proliferation	[16,88,103]
Adipose-derived MSC (AD-MSC)	VEGF, miR-146a, MMP-2, ECM proteins	Pro-angiogenic, anti-fibrotic, immunomodulatory	Inhibited epithelial–mesenchymal transition, restored limbal/corneal phenotypes, reduced ocular surface inflammation	[104,105,106,107,108]
Corneal Stromal MSC (Cornea-MSC)	sFLT-1, PEDF, bFGF, TGFβ3	Anti-angiogenic, stromal regeneration	Prevented corneal neovascularization, promoted stromal matrix deposition, keratocyte differentiation	[109,110,111]
